# Supplementary material for: Altered microRNA Transcriptome in Cultured Human Liver Cells upon Infection with Ebola Virus
Source: Int J Mol Sci. 2021 Apr 6;22(7):3792. doi: 10.3390/ijms22073792 (PMC8038836; doi:10.3390/ijms22073792)
Supplement: Supplementary file 1 [file ijms-22-03792-s001.zip › Supplementary_File/C_ GO_Analysis_Results/16-30nt_go_Mayinga-96h-Huh7_vs_Control-96h-Huh7_down.mature_mirna_targets/GeneOntology_FoldEnrichment.pdf]

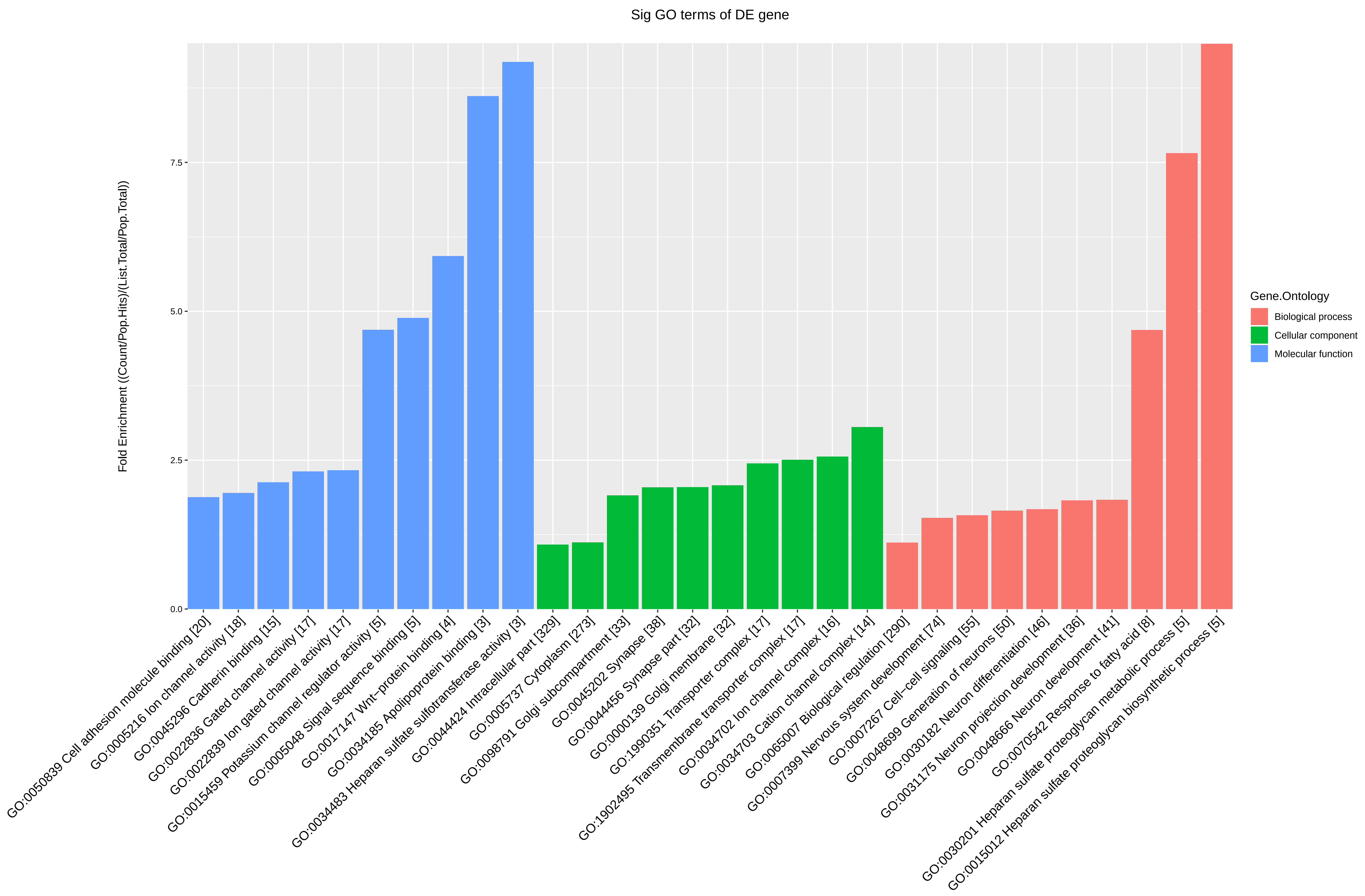

Sig GO terms of DE gene
Fold Enrichment ((Count/Pop.Hits)/(List.Total/Pop.Total))
0.0
2.5
5.0
7.5
Gene.Ontology
Biological process
Cellular component
Molecular function
GO:0050839 Cell adhesion molecule binding [20]
GO:0005216 Ion channel activity [18]
GO:0045296 Cadherin binding [15]
GO:0022836 Gated channel activity [17]
GO:0022839 Ion gated channel activity [17]
GO:0015459 Potassium channel regulator activity [5]
GO:0005048 Signal sequence binding [5]
GO:0017147 Wnt−protein binding [4]
GO:0034185 Apolipoprotein binding [3]
GO:0034483 Heparan sulfate sulfotransferase activity [3]
GO:0044424 Intracellular part [329]
GO:0005737 Cytoplasm [273]
GO:0098791 Golgi subcompartment [33]
GO:0045202 Synapse [38]
GO:0044456 Synapse part [32]
GO:0000139 Golgi membrane [32]
GO:1990351 Transporter complex [17]
GO:1902495 Transmembrane transporter complex [17]
GO:0034702 Ion channel complex [16]
GO:0034703 Cation channel complex [14]
GO:0065007 Biological regulation [290]
GO:0007399 Nervous system development [74]
GO:0007267 Cell−cell signaling [55]
GO:0048699 Generation of neurons [50]
GO:0030182 Neuron differentiation [46]
GO:0031175 Neuron projection development [36]
GO:0048666 Neuron development [41]
GO:0070542 Response to fatty acid [8]
GO:0030201 Heparan sulfate proteoglycan metabolic process [5]
GO:0015012 Heparan sulfate proteoglycan biosynthetic process [5]
